# Clinical Characteristics and Predictive Factors of Immune-Mediated Cholangitis: A Large Single-Center Retrospective Observational Study

**DOI:** 10.3390/cancers18040685

**Published:** 2026-02-19

**Authors:** Noriaki Iijima, Yasutaka Ishii, Shinya Nakamura, Juri Ikemoto, Masaru Furukawa, Yumiko Yamashita, Risa Nomura, Shin Ohtagaki, Yoshihiro Tanaka, Morihito Okada, Noboru Hattori, Sachio Takeno, Nobuyuki Hinata, Akio Tanaka, Wataru Okamoto, Hideki Ohdan, Souichi Yanamoto, Tomonao Aikawa, Ken Yamaguchi, Shinya Takahashi, Tatsuo Ichinohe, Yuji Murakami, Masataka Tsuge, Shiro Oka

**Affiliations:** 1 Department of Gastroenterology, Graduate School of Biomedical and Health Sciences, Hiroshima University, 1-2-3 Kasumi, Minami-ku, Hiroshima 734-8551, Japan; iijiman@hiroshima-u.ac.jp (N.I.);; 2 Department of Surgical Oncology, Research Institute for Radiation Biology and Medicine, Hiroshima University, 1-2-3 Kasumi, Minami-ku, Hiroshima 734-8551, Japan; 3 Department of Molecular and Internal Medicine, Graduate School of Biomedical and Health Sciences, Hiroshima University, 1-2-3 Kasumi, Minami-ku, Hiroshima 734-8551, Japan; 4 Department of Otorhinolaryngology, Head and Neck Surgery, Graduate School of Biomedical and Health Sciences, Hiroshima University, 1-2-3 Kasumi, Minami-ku, Hiroshima 734-8551, Japan; takeno@hiroshima-u.ac.jp; 5 Department of Urology, Graduate School of Biomedical and Health Sciences, Hiroshima University, 1-2-3 Kasumi, Minami-ku, Hiroshima 734-8551, Japan; 6 Department of Dermatology, Graduate School of Biomedical and Health Sciences, Hiroshima University, 1-2-3 Kasumi, Minami-ku, Hiroshima 734-8551, Japan; 7 Cancer Treatment Center, Hiroshima University Hospital, 1-2-3 Kasumi, Minami-ku, Hiroshima 734-8551, Japan; 8 Department of Gastroenterological and Transplant Surgery, Graduate School of Biomedical and Health Science, Hiroshima University, 1-2-3 Kasumi, Minami-ku, Hiroshima 734-8551, Japan; 9 Department of Oral Oncology, Graduate School of Biomedical and Health Science, Hiroshima University, 1-2-3 Kasumi, Minami-ku, Hiroshima 734-8551, Japan; syana@hiroshima-u.ac.jp; 10 Program of Dentistry, Department of Oral and Maxillofacial Surgery, Graduate School of Biomedical and Health Sciences, Hiroshima University, 1-2-3 Kasumi, Minami-ku, Hiroshima 734-8551, Japan; 11 Department of Obstetrics and Gynecology, Graduate School of Biomedical and Health Sciences, Hiroshima University, 1-2-3 Kasumi, Minami-ku, Hiroshima 734-8551, Japan; 12 Department of Surgery, Graduate School of Biomedical and Health Sciences, Hiroshima University, 1-2-3 Kasumi, Minami-ku, Hiroshima 734-8551, Japan; 13 Department of Hematology and Oncology, Research Institute for Radiation Biology and Medicine, Hiroshima University, 1-2-3 Kasumi, Minami-ku, Hiroshima 734-8551, Japan; 14 Department of Radiation Oncology, Graduate School of Biomedical and Health Sciences, Hiroshima University, 1-2-3 Kasumi, Minami-ku, Hiroshima 734-8551, Japan; yujimura@hiroshima-u.ac.jp; 15 Liver Center, Hiroshima University Hospital, 1-2-3 Kasumi, Minami-ku, Hiroshima 734-8551, Japan

**Keywords:** immune checkpoint inhibitors, immune-mediated adverse events, liver injury, cholangitis, eosinophils, C-reactive protein

## Abstract

Background: Immune-mediated cholangitis (IMC) is a rare complication of immune checkpoint inhibitor (ICI) therapy, and its clinical characteristics and prognostic implications remain unclear. This study aimed to clarify the characteristics, risk factors, and outcomes of IMC compared with non-cholangitis cases of immune-mediated hepatotoxicity (IMH). Methods: In this single-center retrospective study, 1332 patients who received ICIs between 2014 and 2023 were analyzed. IMH was diagnosed based on liver enzyme elevation and exclusion of other liver diseases, while IMC was identified through characteristic imaging findings. Baseline factors, clinical presentations, treatment responses, and overall survival (OS) were evaluated. Multivariate analysis identified IMC risk and IMH prognostic factors. Results: Among the cohort, 81 (6.1%) patients had IMH, including 10 (0.8%) with IMC. Baseline eosinophil count > 270/μL (odds ratio [OR] 10.33, *p* = 0.004) and C-reactive protein (CRP) levels > 0.8 mg/dL (OR 6.260, *p* = 0.027) were independent predictors of IMC. IMC was associated with delayed onset, cholestatic liver injury, abdominal pain, and neutrophil-predominant inflammation. In prognostic analysis, IMC was not associated with OS. However, cholestatic liver injury (hazard ratio [HR] 2.318, *p* = 0.023) and neutrophil-to-lymphocyte ratio (NLR) ≥ 4.0 (HR 3.622, *p* = 0.001) were independent predictors of poor OS. Bile duct imaging abnormalities before or after onset were common in patients with treatment-resistant IMC. Conclusions: Baseline eosinophil count and CRP may help predict IMC. While IMC was not a prognostic factor, cholestatic injury and high NLR were associated with worse outcomes. IMC exhibits distinct clinical features, and radiologic findings may support earlier diagnosis and management.

## Simple Summary

Immune checkpoint inhibitors are widely used cancer treatments, but they can sometimes cause liver and bile duct injury. Among these reactions, immune-mediated cholangitis is rare and difficult to diagnose early because symptoms are often unclear. In this study, we examined more than 1300 treated patients to clarify who is more likely to develop this condition and how it affects outcomes. We found that people with higher levels of eosinophils or C-reactive protein before treatment were more likely to develop bile duct injury. We also learned that patients with a cholestatic pattern of liver injury or a high neutrophil-to-lymphocyte ratio tended to have poorer survival. Imaging of the bile ducts often showed abnormalities before or after symptoms appeared and may help identify patients who need closer monitoring. These findings may support earlier diagnosis and more tailored management for patients receiving immunotherapy.

## 1. Introduction

Immune checkpoint inhibitors (ICIs) enhance antitumor immunity by inhibiting cytotoxic T-lymphocyte-associated antigen-4 (CTLA-4), programmed death-1 receptor (PD-1), and its ligand (PD-L1), thereby promoting T-cell responses against tumor-specific antigens [1]. The efficacy of ICIs has been approved for various diseases, and their indications are rapidly expanding [1,2]. However, excessive immune activation or autoimmune reactions can lead to immune-mediated adverse events (imAEs), which are autoimmune side effects impacting various organ systems and pose significant management challenges [1,2,3].

Among imAEs, immune-mediated hepatotoxicity (IMH) is a major concern, occurring in approximately 5–10% of patients undergoing ICI monotherapy and in 25–30% of those receiving combination therapy with anti-PD(L)-1 and anti-CTLA-4 antibodies [3]. Immune-mediated cholangitis (IMC), a rare subtype of IMH, is a secondary sclerosing cholangitis characterized by non-obstructive biliary dilatation and bile duct wall thickening that develops following ICI administration [4]. It was first reported by Gelsomino et al. in 2017 [5], and subsequent reports have increased in number [6,7]. However, IMC remains extremely rare, with an incidence of 0.05–0.76% [8,9,10,11,12,13], and many of its clinical features remain unclear. In contrast to other imAEs, corticosteroid response rates are reported to be poor, ranging from 11.5% to 40.0% [13,14,15]. Moreover, biliary drainage is frequently reported as ineffective [13,14,16], highlighting the need for the development of appropriate diagnostic and therapeutic strategies.

Previous studies by Takinami et al. and Yamamoto et al. have reported clinical differences between IMC and non-cholangitis IMH cases, classified based on imaging findings [12,17]. These studies suggest that the two conditions differ regarding onset timing, liver enzyme patterns, and treatment responsiveness, underscoring the importance of imaging evaluations for early diagnosis. While the clinical characteristics of IMC are gradually being elucidated, its extremely low incidence and nonspecific symptoms make it prone to being overlooked in clinical practice. To enable timely and accurate diagnosis of IMC and to predict treatment response, it is essential to identify IMC-specific clinical features and risk factors. Several clinical factors have been reported as predictors of IMH, including the use of ipilimumab, fever within 24 h after the first ICI dose [10], female sex, a history of imAEs [18], high ICI dosage, and combination therapy with ipilimumab and nivolumab [19]. However, no specific predictive factors for developing IMC have been reported to date.

Immune-mediated cholangitis most commonly presents with a cholestatic pattern of liver injury; however, laboratory abnormalities alone are often insufficient for diagnosis, and imaging-based evaluation using modalities such as contrast-enhanced CT or MRCP is essential. Because IMC is extremely rare and its characteristic features are not widely recognized, diagnosis can be challenging in clinical practice. Moreover, the mechanisms underlying IMC development remain unclear, and optimal therapeutic strategies have yet to be established.

In this study, we aimed to classify IMC and non-cholangitis IMH among patients with IMH within a large cohort of over 1300 patients treated with ICIs and compare their clinical features, risk factors, treatment responsiveness, and prognosis. Through this analysis, we sought to identify IMC-specific biomarkers and clinical characteristics and provide a foundation for early diagnosis and personalized treatment strategies.

## 2. Materials and Methods

### 2.1. Study Population

Using a data warehouse, we retrospectively analyzed data from 1332 patients who received ICIs at Hiroshima University Hospital between January 2014 and December 2023. The study protocol adhered to the Declaration of Helsinki and was approved by the Ethics Committee of Hiroshima University Hospital (Approval No.: E2022-0232).

### 2.2. Clinical Assessment

The ICIs used in this study were as follows:

- Anti-PD-1 antibodies (aPD-1 Ab): nivolumab and pembrolizumab;
- Anti-PD-L1 antibodies (aPD-L1 Ab): atezolizumab, durvalumab, and avelumab;
- Anti-CTLA4 antibodies (aCTLA-4 Ab): ipilimumab and tremelimumab.

It was administered as a single agent or in combination with two agents, depending on the indication for each disease. After ICI administration, we evaluated patients’ general physical condition and repeated blood test results at least every 4 weeks according to institutional practice and the discretion of the attending physicians. Hematological and biochemical biomarkers were evaluated at predefined time points: baseline values were defined as laboratory data obtained immediately before the initiation of immune checkpoint inhibitor therapy, and values at onset were defined as those measured at the time of immune-mediated hepatotoxicity diagnosis. Although laboratory tests were serially performed during ICI treatment, dynamic longitudinal changes in biomarkers were not analyzed, as the primary objective was to identify predictive factors based on predefined baseline and onset values. Adverse events occurring after initiating immunotherapy were assessed according to the Common Terminology Criteria for Adverse Events version 5.0.

**Figure 1:**
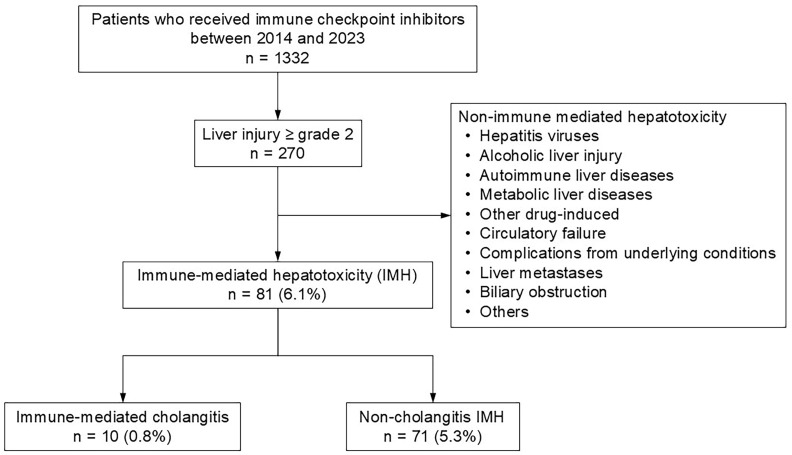
Flowchart showing the selection of patients with immune-mediated hepatotoxicity and immune-mediated cholangitis from the total cohort.

Patients whose aspartate transaminase (AST), alanine aminotransferase (ALT), alkaline phosphatase (ALP), or gamma-glutamyl transferase (GGT) levels were elevated to Grade 2 or higher were identified. To ensure that immune-mediated hepatotoxicity was appropriately distinguished from other causes of liver injury, and consistent with the differential diagnostic process recommended for immune-related adverse events, we followed the Japanese diagnostic criteria for ICI-induced liver injury [20]. Accordingly, we excluded patients with other potential causes of liver dysfunction, including hepatitis viruses (hepatitis A, B, or C virus); alcoholic liver injury; autoimmune liver diseases (autoimmune hepatitis, primary biliary cholangitis, or primary sclerosing cholangitis [PSC]); other drug-induced liver injuries, including those due to concomitant agents or cases in which attribution to ICIs was unclear; metabolic liver diseases (e.g., metabolic dysfunction-associated steatotic liver disease); circulatory failure; complications from underlying conditions; liver metastases; biliary obstruction; or cases in which the cause of liver dysfunction was unknown. We defined IMH as patients excluding these factors (Figure 1).

Among patients with IMH, we defined IMC as those showing imaging findings of extrahepatic bile duct changes (localized dilation without obstruction and/or diffuse thickening of the bile duct wall) and/or intrahepatic bile duct (IHBD) changes, including PSC-like strictures [21], as assessed by contrast-enhanced CT and/or magnetic resonance cholangiopancreatography (MRCP). The patterns of liver dysfunction were classified based on previous reports [22,23] as follows:

- Hepatocellular type: ALT alone elevated to more than five times the upper limit of normal (ULN), or the serum activity ratio of ALT to ALP (expressed as multiples of ULN) = R ≥ 5.
- Cholestatic type: ALP alone elevated to more than two times the ULN, or R < 2.
- Mixed type: R ≥ 2 but <5.

Patients whose AST, ALT, ALP, and GGT levels improved to Grade 1 or below and remained stable were classified as “improved.” Those who initially declined to Grade 1 or below but subsequently increased to Grade 2 or higher were classified as “relapse.” Overall survival (OS) was assessed as the period from the start of ICI treatment to the date of death.

### 2.3. Treatment for IMH

Treatment for IMH primarily followed the guidelines of the European Society for Medical Oncology and the American Association for the Study of Liver Diseases [2,3], with the general approach described below:

- For Grade 2 IMH, ICI therapy was withheld, and liver enzymes were regularly monitored. If liver enzyme levels persisted or worsened, prednisolone (PSL) at 0.5–1 mg/kg/day or an equivalent dose of corticosteroids was administered.
- For Grade 3 or higher, ICI therapy was discontinued, hospitalization was considered, and PSL at 1–2 mg/kg/day or an equivalent dose of corticosteroids was administered.
- For cases refractory to the above treatment, mycophenolate mofetil (MMF) at 2 g/day was administered.

No clear guidelines existed for using ursodeoxycholic acid (UDCA); its administration (300–600 mg/day) was left to the discretion of the attending physician.

### 2.4. Outcomes

The primary outcomes of this study were the clinical characteristics, risk factors, and treatment responses associated with IMC among patients who had IMH following ICI therapy. Secondary outcomes included the identification of prognostic factors for OS among patients with IMH. Multivariate analysis was performed to evaluate the impact of clinical variables, including the presence of IMC, liver injury pattern, inflammatory markers, and treatment response.

### 2.5. Statistical Analysis

Statistical analyses were performed using JMP Pro 18.0.0 (SAS Institute Inc., Cary, NC, USA). Continuous variables, expressed as medians (interquartile range, IQR), were compared using the Kruskal–Wallis test. Categorical variables, expressed as numbers (percentages), were compared between groups using the χ^2^ test or Fisher’s exact test. Due to the extreme rarity of IMC, a direct comparison with the entire non-cholangitis population (n = 1322) was not feasible. To minimize potential confounding and improve robustness, we adopted a two-step analytical approach: first, we compared patients with and without immune-mediated hepatotoxicity (IMH), and second, within the IMH group, we compared patients with IMC and those with non-cholangitis IMH. This strategy facilitated clinically meaningful comparisons while reducing heterogeneity. Logistic regression analysis was performed to identify risk factors for IMC, and Cox proportional hazards models were used to assess prognostic factors for IMH. To further limit confounding and avoid overfitting given the small number of IMC events, only variables with a *p* value < 0.01 in univariate analyses were included in multivariate models. In addition, Firth penalized logistic regression analysis, which provides bias-reduced estimates in small datasets [24,25], was performed. For continuous variables used in the logistic and Cox regression models, optimal cutoff values were determined using receiver operating characteristic curve analysis, based on the maximum Youden Index. Survival analysis for IMH was conducted using the Kaplan–Meier method, and differences between groups were compared using the log-rank test. Two-tailed *p* values < 0.05 were considered statistically significant.

## 3. Results

### 3.1. Baseline Characteristics Before ICI Treatment

IMH was identified in 81 (6.1%) patients, of whom 10 (0.8% of the total cohort) were diagnosed with IMC (Figure 1). Baseline clinical characteristics of patients with and without IMH before initiating ICI therapy are summarized in Table 1. Overall, patients with IMH differed from those without IMH in primary disease distribution and immune checkpoint inhibitor regimen. In particular, combination therapy with anti–PD-1 plus anti–CTLA-4 antibodies was more frequent in the IMH group, whereas anti–PD-L1 monotherapy was more common in the IMH-negative group.

A similar comparison was conducted between IMC and non-cholangitis IMH patients (Table 2). Regarding ICI regimens, anti–PD-1 antibody monotherapy tended to be more frequent in the IMC group (90.0% vs. 56.3%, *p* = 0.080), whereas combination therapy with anti–PD-1 and anti–CTLA-4 antibodies tended to be more common in the non-cholangitis IMH group (0% vs. 32.4%, *p* = 0.055). No notable differences were observed between the two groups in age, sex, performance status, primary disease, or the use of concomitant cytotoxic chemotherapy or molecular targeted agents.

With respect to baseline laboratory findings before treatment initiation, the IMC group showed significantly higher eosinophil counts and CRP levels (*p* = 0.021 and 0.027, respectively). In addition, WBC and neutrophil counts were numerically higher in the IMC group, although these differences did not reach statistical significance (*p* = 0.081 and 0.088, respectively).

### 3.2. Risk Factors for IMC Onset

Logistic regression analysis was subsequently performed to identify factors associated with IMC development (Table 3). In the univariate analysis, anti–PD-1 antibody monotherapy, elevated baseline eosinophil count, and elevated baseline CRP level were associated with IMC occurrence. In the multivariate analysis including variables with *p* < 0.01 in univariate analysis, baseline eosinophil count > 270/μL (odds ratio [OR]: 10.33, 95% confidence interval [CI]: 2.092–77.10, *p* = 0.004) and baseline CRP level > 0.8 mg/dL (OR: 6.260, 95% CI: 1.229–47.25, *p* = 0.027) were identified as independent risk factors for IMC onset. Similar results were obtained using Firth penalized logistic regression analysis (Table 4).

### 3.3. Comparison of Clinical Characteristics and Treatment Outcomes Between IMC and Non-Cholangitis IMH

The clinical characteristics after IMH onset were compared between the IMC and non-cholangitis IMH groups (Table 5). The number of ICI cycles until IMH onset was significantly higher in the IMC group than in the non-cholangitis IMH group (median, 4 vs. 2 cycles; *p* = 0.023). Regarding the type of liver injury, the cholestatic pattern was significantly more frequent in the IMC group (80.0% vs. 31.0%, *p* = 0.004), whereas the hepatocellular pattern was observed only in the non-cholangitis IMH group (0% vs. 43.7%, *p* = 0.011). Abdominal symptoms were present in all patients with IMC, compared with 66.2% of patients with non-cholangitis IMH. In particular, abdominal pain was significantly more common in the IMC group (40.0% vs. 11.3%, *p* = 0.037). There were no significant differences between the two groups in the grade of liver injury or the incidence of extrahepatic imAEs. Laboratory findings at the onset of IMH showed significantly higher white blood cell count, C-reactive protein, alkaline phosphatase, gamma-glutamyl transferase, and neutrophil-to-lymphocyte ratio in the IMC group (*p* < 0.001, 0.008, <0.001, 0.002, and 0.004, respectively). Regarding treatment, corticosteroids, ursodeoxycholic acid, and mycophenolate mofetil were used in both groups. Improvement of immune-mediated hepatotoxicity was observed in five patients (50.0%) in the IMC group and in 48 patients (67.6%) in the non-cholangitis IMH group, with no statistically significant difference between the groups.

### 3.4. Prognostic Factors for IMH

Cox proportional hazards analysis was performed to identify prognostic factors in patients with IMH (Table 6). Univariate analysis was conducted considering variables with *p* < 0.01 as statistically significant, and the following were identified as significant prognostic factors: cholestatic-type liver injury, WBC count ≥8000/μL at onset, NLR ≥4.0 at onset, and improvement of IMH. Multivariate analysis identified the cholestatic type of liver injury (hazard ratio [HR]: 2.318, 95% CI: 1.125–4.777, *p* = 0.023) and NLR ≥4.0 at onset (HR: 3.622, 95% CI: 1.683–7.791, *p* = 0.001) as independent prognostic factors.

**Figure 2:**
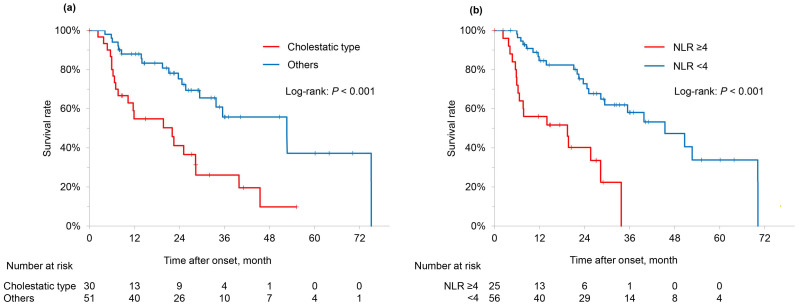
Kaplan–Meier analysis of overall survival in patients with IMH. (**a**) Comparison of overall survival (OS) between patients with cholestatic-type liver injury and those with other types (hepatocellular or mixed-type liver injury). The cholestatic group exhibited a significantly shorter median survival time (MST) (22.1 months) than the non-cholestatic group (52.6 months) (log-rank test, *p* < 0.001). (**b**) Comparison of OS stratified based on the neutrophil-to-lymphocyte ratio (NLR) at the onset of liver injury. Patients with NLR ≥ 4.0 had significantly shorter MST (19.5 months) than those with NLR < 4.0 (45.5 months) (log-rank test, *p* < 0.001).

Kaplan–Meier analysis was also performed to evaluate OS in patients with IMH (Figure 2). When stratified by liver injury pattern, patients with cholestatic-type liver injury showed significantly shorter survival than those with non-cholestatic injury, with median survival times of 22.1 and 52.6 months, respectively. Similarly, patients with NLR ≥4.0 at IMH onset had significantly shorter survival than those with NLR < 4.0 (median survival time: 19.5 vs. 45.5 months).

### 3.5. Clinical Characteristics and Imaging Findings of Patients with IMC

The detailed clinical characteristics and clinical course of the 10 patients with IMC are summarized in Table 7, while detailed imaging findings before onset, at onset, and after treatment are presented in Table 8. Serum IgG4 levels were measured in seven patients, and no elevation was observed in any case. At IMC onset, contrast-enhanced CT was performed in all patients, and MRCP was performed in eight patients; accordingly, the diagnosis of IMC was primarily based on contrast-enhanced CT and MRCP findings. Clinically, treatment responses varied across patients, with biochemical improvement observed in five cases, whereas the remaining patients experienced relapse or showed no improvement despite treatment. At onset, most patients demonstrated a cholestatic pattern of liver injury accompanied by characteristic bile duct abnormalities, including common bile duct involvement and/or intrahepatic bile duct changes. Importantly, evaluation of serial imaging findings revealed that persistent bile duct abnormalities after treatment were frequently observed, particularly among patients without biochemical improvement. In contrast, normalization or resolution of imaging abnormalities was more commonly seen in patients who achieved biochemical improvement. These findings suggest a potential association between radiologic persistence and treatment resistance in IMC.

## 4. Discussion

In this study, we comprehensively investigated the clinical characteristics, risk factors for onset, and prognostic impact of IMC, a rare but distinct imAE associated with ICIs. Among IMH, cholangitis and non-cholangitis liver injury represent clinically heterogeneous entities with different patterns of organ involvement and treatment responsiveness, making their distinction and comparison clinically important. Overall, our findings are largely consistent with previous reports regarding the incidence, clinical presentation, and treatment responsiveness of IMC; however, most earlier studies were descriptive case series, whereas our study provides a direct comparison between IMC and non-cholangitis immune-mediated hepatotoxicity within a large, well-characterized cohort. By categorizing IMH into IMC and non-cholangitis IMH groups and comparing them, we clarified the specific clinical features of IMC and identified potential biomarkers that may facilitate early diagnosis and therapeutic decision-making. Although several case series and reviews of IMC alone have been reported—some with larger sample sizes or multicenter designs [6,7,9,10,12,13,14,15]—comparative analyses between IMC and non-cholangitis IMH remain extremely rare [12,17]. We acknowledge the limitations of this single-center retrospective study, but believe that our comparison between IMC and non-cholangitis IMH provides valuable insights and may serve as a reference for future multicenter studies. In addition, because the number of IMC cases was limited, we performed an additional multivariate analysis using Firth penalized logistic regression to minimize potential bias arising from the small sample. This analysis yielded consistent results, supporting the robustness of our findings regarding baseline eosinophil count and CRP level as independent risk factors for IMC.

One of the principal findings of this study was that elevated baseline eosinophil count ≥270/μL and CRP levels ≥ 0.8 mg/dL were independent predictive factors for IMC onset. To the best of our knowledge, this is the first report to identify IMC-specific risk factors. Elevated eosinophil counts and CRP levels have been reported as risk factors for various other imAEs, including dermatologic, pulmonary, and endocrine disorders [26,27,28,29,30,31,32,33,34]. Yoshikawa et al. also reported that an eosinophil count ≥ 130/μL was associated with an increased risk of IMH [35]. Eosinophils have been implicated in the activation of T cells and the recruitment of tumor-specific CD8^+^ T-cells [27], and it has also been suggested that ICI administration in patients with pre-existing systemic inflammation may promote the production of proinflammatory cytokines and thereby increase the incidence of imAEs [32]. These findings suggest that elevated baseline levels of eosinophils and CRP may reflect a preactivated immune state or a predisposition to develop imAEs. Our findings extend these observations to IMC and suggest that patients with elevated inflammatory markers are more susceptible to hepatobiliary imAEs. Although the underlying mechanisms remain unclear, they are likely multifactorial. Sawada et al. reported eosinophilic infiltration in liver biopsies from a patient with IMC, along with CD8+ T-cell infiltration, indicating pathological overlap with allergic drug-induced liver injury [36]. Other studies have also described infiltration by various inflammatory cells, including neutrophils, eosinophils, lymphocytes, and plasma cells, in liver or bile duct biopsies from IMC cases [8,37,38,39]. Consistently, our study demonstrated elevated WBC counts and CRP levels both before and after imAE onset in patients with IMC, suggesting that not only T lymphocytes but also diverse inflammatory cells are involved in IMC pathogenesis.

Clinically, IMC tended to occur after a higher number of ICI administrations than non-cholangitis IMH cases and was frequently accompanied by cholestatic liver injury and abdominal pain, which is consistent with previous reports describing a high prevalence of abdominal symptoms and a predominance of cholestatic liver injury in IMC. These features are consistent with those of previous reports showing a median of 3–6 ICI administrations at IMC onset [6,12,13,14,15,17], cholestatic injury in 50.0–100.0% [7,12,13,17], and abdominal pain in 30.0–87.5% of patients [12,13,14]. Yamamoto et al. also reported that CRP and NLR levels were significantly higher in patients with IMC than in those without cholangitis IMH [12]. Collectively, these findings suggest that IMC is characterized as a “delayed-onset, cholestatic liver injury with abdominal pain and a neutrophil-dominant inflammatory profile.”

Regarding the type of ICI used, IMC was more frequently observed in patients treated with anti-PD-1 monotherapy, whereas those without cholangitis IMH more often received anti-PD-1 plus anti-CTLA-4 combination therapy. This trend is also consistent with that of previous studies, where IMC was predominantly associated with anti-PD-1 monotherapy [6,7,12,13,14,15]. Indeed, 75.0–100.0% of IMC cases in prior reports were treated with anti-PD-1 antibodies alone, suggesting a possible association between this agent class and IMC development. Conversely, anti-CTLA-4 antibodies and combination therapies have been implicated as risk factors for IMH [10,18]. Within the immune checkpoint pathways, CTLA-4 primarily regulates initial T-cell activation in lymph nodes, whereas PD-1 modulates later responses in peripheral tissues. Anti-PD-1 antibodies block both PD-L1 and PD-L2 interactions, while anti-PD-L1 antibodies do not affect PD-1/PD-L2 signaling but instead inhibit the PD-L1/B7-1 interaction [40,41,42]. These molecular differences may influence the timing and mechanism of cholangitis development. Although Stein et al. reported an association between PD-L1 expression on biliary epithelial cells and cholangitis in a mouse model, further mechanistic studies are limited and warranted [43]. These findings extend previous observations by demonstrating this trend in a comparative analysis against non-cholangitis IMH, rather than in IMC case series alone.

Given the neutrophil-dominant inflammatory profile observed in IMC in our cohort, we incorporated neutrophil-related indices into the prognostic analysis. Although several composite indices derived from hematologic parameters have been proposed as prognostic biomarkers, we intentionally focused on simple and routinely available markers, as composite scores incorporating platelet count or serum albumin may be substantially influenced by liver dysfunction itself and complicate interpretation in immune-mediated hepatobiliary injury. In the analysis of prognostic factors among patients with IMH, the presence of IMC was not significantly associated with OS. However, cholestatic liver injury and elevated NLR were independently associated with a poor prognosis. Holmstroem et al. demonstrated that cholestatic liver injury was a negative prognostic factor in a prospective interventional study [44]. Additionally, high NLR after imAE onset has been reported to be associated with poor outcomes [45]. These parameters are readily available from routine blood tests and may serve as practical prognostic biomarkers in clinical settings. While IMC was frequently associated with a cholestatic pattern of liver injury, which was identified as an independent predictor of poor prognosis, it was not a significant prognostic factor in the multivariate analysis. This discrepancy may be attributable to the small number of IMC cases (n = 10), which limited the statistical power to detect a potential prognostic impact. Given that reports on the prognostic relevance of IMC remain insufficient, these findings suggest that the pattern of liver injury, particularly the cholestatic type, has a greater influence on clinical outcomes than the diagnostic category of IMC. Further accumulation of IMC cases and prospective studies are warranted to clarify its prognostic implications. Parameters reflecting liver synthetic function, such as serum albumin levels and coagulation markers, are essential for monitoring the severity of liver injury and guiding clinical management. However, these variables are strongly influenced by liver dysfunction itself and therefore may primarily reflect disease severity rather than serving as predictive or prognostic biomarkers for immune-mediated cholangitis. Accordingly, in the present analysis, we focused on inflammatory markers with potential relevance to immune activation, while acknowledging the clinical importance of liver function parameters for toxicity grading and patient management.

Furthermore, our study evaluated imaging changes before and after IMC onset. In five of the 10 patients (50.0%), imaging abnormalities such as bile duct dilation and wall thickening were observed before the biochemical elevation of liver enzymes. In seven (70.0%) patients, such abnormalities persisted after treatment. These findings were more common among non-responders, indicating that persistent imaging abnormalities may be associated with treatment resistance. Regarding the persistence of bile duct imaging abnormalities after treatment, one possible explanation is the prolonged immunologic effect of immune checkpoint inhibitors, a phenomenon known as delayed immune-related events (DIRE) [46]. Immune checkpoint inhibitors can exert sustained immune activation even after treatment discontinuation, and immune-related adverse events have been reported to occur or persist long after cessation of therapy. Accordingly, ongoing immune-mediated injury to the biliary epithelium may persist despite biochemical improvement, resulting in prolonged or residual radiologic abnormalities. Although some of the imaging findings observed before IMC onset were atypical, when considered together with elevated CRP or eosinophil levels, they may contribute to earlier detection of IMC. Persistent imaging abnormalities following clinical improvement may indicate ongoing immune activity in the biliary system, underscoring the importance of long-term radiologic monitoring.

Currently, standardized treatment strategies and guidelines for ICI rechallenge in IMC remain undefined. In our cohort, all patients discontinued ICIs, and treatment with corticosteroids and UDCA was initiated; however, the overall response rate was limited. Notably, patients with pre-treatment imaging abnormalities and persistent post-treatment changes tended to be steroid-refractory. UDCA may have potential efficacy in cholestatic liver injury [6,47,48,49]; however, due to heterogeneous treatment approaches in our study, further research is needed to establish an optimal therapeutic strategy.

This study had some limitations. First, given the extreme rarity of IMC, this was a single-center retrospective study involving a small number of patients with IMC, which limited the statistical power. To minimize potential confounding, we performed a two step analysis: first comparing patients with and without IMH and then comparing IMC and non-cholangitis IMH cases. Second, imaging assessment and treatment strategies were not standardized, raising the possibility that some IMC cases may have been overlooked. Patients in whom it was difficult to distinguish ICI-induced liver injury from that caused by concomitant treatment were excluded, possibly leading to an underestimation of the actual number of IMC/IMH cases. Third, histological evaluation was not obtained in all cases—biopsies were performed in only four of the 10 patients with IMC. In the remaining patients, biopsy was deemed unnecessary due to mild symptoms, prior improvement, or lack of patient consent. Although CD8+ T-cell infiltration is considered a hallmark of IMC [4], many patients can be adequately diagnosed based on clinical course and imaging findings, and previous studies have cautioned that histological evaluation should be performed judiciously [50,51]. Therefore, a biopsy may not be essential in selected patients.

## 5. Conclusions

In conclusion, elevated baseline eosinophil counts and CRP levels may serve as useful predictors of IMC onset, while cholestatic liver injury and elevated NLR may be clinically important indicators of poor prognosis. IMC has distinct clinical and immunologic characteristics and may be less responsive to conventional steroid therapy. Persistent imaging abnormalities may indicate ongoing immune activation and necessitate prolonged monitoring. From a clinical perspective, these readily available laboratory markers may help identify patients who require closer monitoring during immune checkpoint inhibitor therapy. In addition, bile duct imaging abnormalities observed before or after biochemical onset may support earlier diagnosis and provide insight into treatment resistance. Future prospective, multicenter studies are warranted to validate these findings and to establish standardized diagnostic and therapeutic strategies for immune-mediated cholangitis. This study contributes to a better understanding of this rare entity and may aid in the early diagnosis, risk stratification, and personalized management of patients receiving ICIs.

## Figures and Tables

**Table 1:** Comparison of baseline characteristics between patients with and without immune-mediated hepatotoxicity.

Factors	IMH Positive (*n* = 81)	IMH Negative (*n* = 1251)	*p* Value
Age (years)	68 (56.5–74)	70 (62–76)	0.030
Sex (Male/Female)	49/32	889/362	0.059
ECOG PS (0/≥1)	65 (80.3)/16 (19.8)	918 (73.4)/333 (26.6)	0.194
Primary disease			<0.001
Non-small cell lung cancer	21 (25.9)	373 (29.8)	
Renal cell cancer	15 (18.5)	139 (11.1)	
Malignant melanoma	13 (16.1)	79 (6.3)	
Head-and-neck cancer	10 (12.4)	178 (14.2)	
Breast cancer	5 (6.2)	13 (1.0)	
Gastric cancer	5 (6.2)	64 (5.1)	
Esophageal cancer	3 (3.7)	81 (6.5)	
Hepatocellular carcinoma	3 (3.7)	162 (13.0)	
Others	6 (7.4)	162 (13.0)	
Type of ICI			
aPD-1 Ab	49 (60.5)	858 (68.6)	0.140
aPDL-1 Ab	7 (8.6)	258 (20.6)	0.006
aCTLA4 Ab	1 (1.2)	10 (0.8)	0.500
aPD-1 Ab + aCTLA4 Ab	23 (28.4)	76 (6.1)	<0.001
aPDL-1 Ab + aCTLA4 Ab	2 (2.5)	53 (4.2)	0.770
Combined use with CTX or MTT	25 (30.9)	340 (27.2)	0.520
Baseline laboratory data			
WBC (/μL)	6580 (5470–8320)	5930 (4590–7550)	0.005
ANC (/μL)	4210 (3150–5700)	3840 (2830–5400)	0.051
ALC (/μL)	1470 (1170–1790)	1230 (870–1650)	<0.001
AEC (/μL)	180 (90–270)	120 (60–230)	0.009
PLT (×10^3^/μL)	245 (203–301)	224 (167–289)	0.014
CRP (mg/dL)	0.36 (0.11–1.34)	0.41 (0.11–2.10)	0.027
AST (×ULN)	0.70 (0.57–0.95)	0.70 (0.55–0.93)	0.813
ALT (×ULN)	0.55 (0.39–0.77)	0.43 (0.31–0.67)	0.002
ALP (×ULN)	0.76 (0.59–1.05)	0.76 (0.59–1.00)	0.692
GGT (×ULN)	0.58 (0.41–1.02)	0.57 (0.34–1.16)	0.469
NLR	2.94 (2.18–3.92)	3.21 (2.09–5.13)	0.197
PLR	172.8 (125.0–214.0)	179.6 (123.0–274.5)	0.225
Observation period (month)	23.7 (11.1–34.2)	15.0 (6.6–27.2)	0.001

Data are expressed as a number (percentage) or a median (interquartile range). Abbreviations: ECOG PS, Eastern Cooperative Oncology Group performance status; ICI, immune checkpoint inhibitor; aPD-1 Ab, anti-programmed death-1 antibodies; aPDL-1 Ab, anti-programmed death-Ligand 1 antibodies; aCTLA4 Ab, anti-cytotoxic T-lymphocyte-associated antigen-4 antibodies; CTX, cytotoxic chemotherapy; MTT, molecular targeted therapy; WBC, white blood cell count; ANC, absolute neutrophil count; ALC, absolute lymphocyte count; AEC, absolute eosinophil count; PLT, platelet; CRP, C-reactive protein; AST, aspartate aminotransferase; ALT, alanine aminotransferase; ALP, alkaline phosphatase; GGT, gamma-glutamyl transferase; ULN, upper limit of normal; NLR, neutrophil-to-lymphocyte ratio; PLR, platelet-to-lymphocyte ratio.

**Table 2:** Comparison of baseline characteristics between patients with and without immune-mediated cholangitis.

Factors	IMC (*n* = 10)	Non-Cholangitis IMH (*n* = 71)	*p* Value
Age (years)	70.5 (61.25–74.25)	68 (54–74)	0.490
Sex (Male/Female)	7/3	42/29	0.732
ECOG PS (0/≥1)	6 (60)/4 (40)	59 (83.1)/12 (16.9)	0.102
Primary disease			0.704
Non-small cell lung cancer	3 (30)	18 (25.4)	
Renal cell cancer	2 (20)	13 (18.3)	
Malignant melanoma	0	13 (18.3)	
Head-and-neck cancer	2 (20)	8 (11.3)	
Breast cancer	1 (10)	4 (5.6)	
Gastric cancer	1 (10)	4 (5.6)	
Esophageal cancer	1 (10)	2 (2.8)	
Hepatocellular carcinoma	0	3 (4.2)	
Others	0	6 (8.5)	
Type of ICI			
aPD-1 Ab	9 (90)	40 (56.3)	0.080
aPDL-1 Ab	1 (10)	6 (8.5)	1.000
aCTLA4 Ab	0	1 (1.4)	1.000
aPD-1 Ab + aCTLA4 Ab	0	23 (32.4)	0.055
aPDL-1 Ab + aCTLA4 Ab	0	2 (2.8)	1.000
Combined use with CTX or MTT	5 (50)	20 (28.2)	0.271
Baseline laboratory data			
WBC (/μL)	8250 (6510–8970)	6510 (5240–7440)	0.081
ANC (/μL)	5000 (4420–6170)	4110 (3070–5490)	0.088
ALC (/μL)	1630 (1420–1830)	1440 (1140–1820)	0.373
AEC (/μL)	310 (210–590)	170 (80–240)	0.021
PLT (×10^3^/μL)	243 (224–320)	245 (202–300)	0.672
CRP (mg/dL)	1.40 (0.69–3.83)	0.33 (0.10–0.97)	0.027
AST (×ULN)	0.70 (0.57–0.83)	0.70 (0.57–0.97)	0.807
ALT (×ULN)	0.39 (0.28–0.82)	0.57 (0.40–0.76)	0.121
ALP (×ULN)	0.87 (0.56–1.31)	0.76 (0.59–1.00)	0.523
GGT (×ULN)	0.65 (0.37–1.47)	0.56 (0.42–1.03)	0.736
NLR	3.11 (2.74–4.70)	2.83 (2.15–3.90)	0.201
PLR	162 (132–210)	174 (122–214)	0.841
Observation period (month)	10.7 (6.2–34.2)	24.7 (11.9–34.7)	0.164

Data are expressed as a number (percentage) or a median (interquartile range). Abbreviations: ECOG PS, Eastern Cooperative Oncology Group performance status; ICI, immune checkpoint inhibitor; aPD-1 Ab, anti-programmed death- 1 antibodies; aPDL-1 Ab, anti-programmed death-Ligand 1 antibodies; aCTLA4 Ab, anti-cytotoxic T-lymphocyte-associated antigen-4 antibodies; CTX, cytotoxic chemotherapy; MTT, molecular targeted therapy; WBC, white blood cell count; ANC, absolute neutrophil count; ALC, absolute lymphocyte count; AEC, absolute eosinophil count; PLT, platelet; CRP, C-reactive protein; AST, aspartate aminotransferase; ALT, alanine aminotransferase; ALP, alkaline phosphatase; GGT, gamma-glutamyl transferase; ULN, upper limit of normal; NLR, neutrophil-to-lymphocyte ratio; PLR, platelet-to-lymphocyte ratio.

**Table 3:** Univariate and multivariate analysis of risk factors for developing immune-mediated cholangitis.

	IMC (*n* = 10)	Non-Cholangitis IMH (*n* = 71)	Univariate Analysis		Multivariate Analysis	
			Odds Ratio (95% CI)	*p* Value	Odds Ratio (95% CI)	*p* Value
Age at ICI initiation ≥ 60 years	8	48	1.916 (0.437–13.37)	0.411		
Sex (Male)	7	42	1.611 (0.410–7.951)	0.505		
aPD-1 Ab monotherapy	9	39	7.385 (1.287–139.8)	0.022		
Baseline WBC count ≥ 7000/μL	5	26	1.730 (0.443–6.774)	0.421		
Baseline neutrophil count ≥ 5000/μL	5	24	1.958 (0.500–7.690)	0.326		
Baseline eosinophil count ≥ 270/μL	8	14	16.29 (3.615–116.2)	<0.001	10.33 (2.092–77.10)	0.004
Baseline CRP ≥ 0.8 mg/dL	8	19	10.53 (2.383–74.06)	0.001	6.260 (1.229–47.25)	0.027

Abbreviations: ICI, immune checkpoint inhibitor; aPD-1 Ab, anti-programmed death-1 antibodies; WBC, white blood cell; CRP, C-reactive protein; CI, confidence interval.

**Table 4:** Univariate and multivariate Firth penalized logistic regression analysis of risk factors for immune-mediated cholangitis.

	IMC (*n* = 10)	Non-Cholangitis IMH (*n* = 71)	Univariate Analysis		Multivariate Analysis	
			Odds Ratio (95% CI)	*p* Value	Odds Ratio (95% CI)	*p* Value
Age at ICI initiation ≥ 60 years	8	48	0.963 (0.518–1.789)	0.917		
Sex (Male)	7	42	0.949 (0.534–1.690)	0.872		
aPD-1 Ab monotherapy	9	39	1.729 (0.747–4.003)	0.214		
Baseline WBC count ≥ 7000/μL	5	26	1.715 (0.948–3.103)	0.080		
Baseline neutrophil count ≥ 5000/μL	5	24	1.542 (0.858–2.771)	0.153		
Baseline eosinophil count ≥ 270/μL	8	14	3.815 (1.867–7.796)	<0.001	3.693 (1.842–7.409)	<0.001
Baseline CRP ≥ 0.8 mg/dL	8	19	2.344 (1.141–4.817)	0.018	2.226 (1.107–4.476)	0.025

Abbreviations: ICI, immune checkpoint inhibitor; aPD-1 Ab, anti-programmed death-1 antibodies; WBC, white blood cell; CRP, C-reactive protein; CI, confidence interval.

**Table 5:** Clinical characteristics and treatment outcomes after onset of immune-mediated hepatotoxicity.

Factors	IMC (*n* = 10)	Non-Cholangitis IMH (*n* = 71)	*p* Value
Cycles of ICI infusions until onset	4 (3–7.25)	2 (1–4)	0.023
CTCAE Grade (2/3/≧4)	1 (10)/6 (60)/3 (30)	24 (33.8)/39 (54.9)/8 (11.3)	0.278
Type of liver injury			
Hepatocellular	0	31 (43.7)	0.011
Mixed	2 (20)	20 (28.2)	0.720
Cholestatic	8 (80)	22 (31.0)	0.004
Abdominal symptoms			
any symptom	10 (100)	47 (66.2)	0.029
abdominal pain	4 (40)	8 (11.3)	0.037
Multiple imAEs			
≥2 types	5 (50)	43 (60.6)	0.733
≥3 types	1 (10)	11 (15.5)	1.000
Laboratory data at the time of onset			
WBC (/μL)	9660 (8800–11,900)	5550 (4290–7870)	<0.001
ANC (/μL)	7070 (5540–8990)	3560 (2570–5010)	<0.001
ALC (/μL)	1280 (640–1680)	1260 (910–1800)	0.730
AEC (/μL)	590 (160–1240)	150 (60–370)	0.053
PLT (×10^3^/μL)	306 (220–348)	209 (140–321)	0.062
CRP	5.1 (2.9–11.7)	1.3 (0.3–5.1)	0.008
AST (×ULN)	5.5 (3.8–8.8)	4.5 (2.8–6.7)	0.269
ALT (×ULN)	4.7 (2.3–10.7)	5.1 (2.6–10.4)	0.954
ALP (×ULN)	5.2 (3.7–9.43)	1.3 (0.9–2.3)	<0.001
GGT (×ULN)	6.6 (5.7–30.9)	2.8 (1.1–6.3)	0.002
NLR	4.83 (3.71–12.2)	2.58 (1.83–4.23)	0.004
PLR	242.3 (182.5–324.3)	160.3 (112.3–249.8)	0.044
Improvement of IMH	5 (50)	48 (67.6)	0.303
Treatment Drugs (PSL/UDCA/MMF)	7 (70)/9 (90)/2 (20)	40 (56.3)/44 (62.0)/24 (54.6)	
PSL effective	3 (42.9)	30 (75.0)	0.173

Data are expressed as a number (percentage) or a median (interquartile range). Abbreviations: IMC, immune-mediated cholangitis; ICI, immune checkpoint inhibitor; CTCAE, Common Terminology Criteria for Adverse Events; imAE, immune-mediated adverse event; WBC, white blood cell count; ANC, absolute neutrophil count; ALC, absolute lymphocyte count; AEC, absolute eosinophil count; PLT, platelet; CRP, C-reactive protein; AST, aspartate aminotransferase; ALT, alanine aminotransferase; ALP, alkaline phosphatase; GGT, gamma-glutamyl transferase; ULN, upper limit of normal; NLR, neutrophil-to-lymphocyte ratio; PLR, platelet-to-lymphocyte ratio; PSL, prednisolone; UDCA, ursodeoxycholic acid; MMF, mycophenolate mofetil.

**Table 6:** Univariate and multivariate analysis of prognostic factors associated with immune-mediated hepatotoxicity.

	Number	Univariate Analysis		Multivariate Analysis *	
		Hazard Ratio (95% CI)	*p* Value	Hazard Ratio (95% CI)	*p* Value
Age (years)			0.822		
<60	25	Ref.			
≥60	56	1.085 (0.531–2.219)			
Sex			0.292		
Female	32	Ref.			
Male	49	1.444 (0.729–2.859)			
IMC			0.164		
Yes	10	1.974 (0.757–5.152)			
No	71	Ref.			
Cycles of ICI infusions			0.396		
<3	42	Ref.			
≥3	39	1.319 (0.696–2.501)			
CTCAE Grade			0.494		
<3	70	Ref.			
≥4	11	0.733 (0.301–1.785)			
Cholestatic type of liver injury			0.001		0.023
Yes	30	2.959 (1.567–5.587)		2.318 (1.125–4.777)	
No	51	Ref.		Ref.	
Multiple imAEs			0.184		
Yes	48	0.651 (0.346–1.226)			
No	33	Ref.			
Abdominal pain			0.019		
Yes	12	2.804 (1.185–6.634)			
No	59	Ref.			
WBC count at onset (/μL)			0.005		0.470
<8000	58	Ref.		Ref.	
≥8000	23	2.671 (1.347–5.297)		1.348 (0.600–3.030)	
CRP level at onset (mg/dL)			0.142		
<2.0	44	Ref.			
≥2.0	37	1.617 (0.851–3.072)			
NLR at onset			<0.001		0.001
<4.0	56	Ref.		Ref.	
≥4.0	25	3.932 (1.968–7.857)		3.622 (1.683–7.791)	
PLR at onset			0.037		
<180	39				
≥180	42	1.998 (1.043–3.826)			
Improvement of IMH			0.008		0.097
Yes	53	0.424 (0.225–0.800)		0.562 (0.284–1.111)	
No	28	Ref.		Ref.	

* Multivariate analysis was performed using a Cox proportional hazards model including variables with a *p* value < 0.01 in the univariate analysis. Abbreviations: IMC, immune-mediated cholangitis; ICI, immune checkpoint inhibitor; CTCAE, Common Terminology Criteria for Adverse Events; imAE, immune-mediated adverse event; WBC, white blood cell; CRP, C-reactive protein; NLR, neutrophil-to-lymphocyte ratio; PLR, platelet-to-lymphocyte ratio; IMH, immune-mediated hepatotoxicity; CI, confidence interval.

**Table 7:** Clinical characteristics and clinical course of patients with immune-mediated cholangitis.

No	Age /Sex	Primary Site	ICI	ICI Cycles	Grade	Liver Injury Pattern	Symptoms	Treatment	Clinical Course
1	71/M	Lung	Nivo	16	4	Cholestatic	Abdominal pain	PSL, UDCA, MMF	Relapse
2	74/M	Lung	Nivo	3	3	Cholestatic	Abdominal pain	PSL, UDCA	Improved
3	77/F	Lung	Pem	3	3	Cholestatic	Appetite loss, jaundice, ascites	UDCA, ENBD	Never improved
4	64/F	Kidney	Pem	5	3	Cholestatic	Abdominal pain, jaundice	PSL, UDCA, MMF, ENBD	Never improved
5	59/M	Kidney	Pem	3	4	Mixed	Fever, jaundice	PSL, UDCA, ENBD	Improved
6	72/M	Hypopharynx	Pem	8	3	Cholestatic	Fever, appetite loss	UDCA	Relapse
7	56/M	Larynx	Pem	2	3	Mixed	Abdominal pain, appetite loss	Observation	Improved
8	62/M	Stomach	Nivo	7	2	Cholestatic	Fever, ascites	PSL, UDCA	Never improved
9	70/M	Esophagus	Pem	3	3	Cholestatic	Nausea, appetite loss	PSL, UDCA	Improved
10	75/F	Breast	Atezo	5	3	Cholestatic	Fever, nausea, appetite loss	PSL, UDCA, ENBD	Improved

Abbreviations: ICI, immune checkpoint inhibitor; Nivo, nivolumab; Pem, pembrolizumab; Atezo, atezolizumab; PSL, prednisolone; UDCA, ursodeoxycholic acid; MMF, mycophenolate mofetil; ENBD, endoscopic nasobiliary drainage.

**Table 8:** Longitudinal imaging characteristics and clinical course of immune-mediated cholangitis.

No	At Onset	Pre-Onset		Post-Treatment		Improvement of IMH **
	Location of Abnormality *	Imaging Abnormality	Location	Imaging Abnormality	Location	
1	CBD involvement IHBD (PSC-like)	Present (3 M before)	CBD (dilation)	Persistent	IHBD (PSC-like)	No
2	CBD involvement IHBD (PSC-like)	Absent	None	Not evaluated	Not evaluated	Yes
3	CBD involvement IHBD (dilatation)	Present (1 M before)	CBD involvement	Persistent	CBD (wall thickening)	No
4	CBD involvement IHBD (PSC-like)	Present (2 M before)	IHBD (dilation)	Persistent	IHBD (PSC-like) CBD (wall thickening)	No
5	CBD involvement IHBD (dilatation)	Absent	None	Resolved	None	Yes
6	CBD involvement	Absent	None	Persistent	CBD (dilation)	No
7	CBD involvement IHBD (dilatation)	Absent	None	Persistent	CBD involvement	Yes
8	CBD involvement IHBD (dilatation)	Present (1 M before)	CBD (dilation)	Persistent	CBD involvement IHBD (dilation)	No
9	CBD involvement	Absent	None	Resolved	None	Yes
10	CBD involvement IHBD (dilatation)	Present (1 M before)	CBD (dilation)	Persistent	CBD (dilation)	Yes

* CBD involvement was defined as bile duct dilatation with wall thickening. ** Biochemical improvement was defined as a sustained decrease in cholestatic liver enzymes after treatment. Abbreviations: CBD, common bile duct; IHBD, intrahepatic bile duct; PSC, primary sclerosing cholangitis.

## Data Availability

The data presented in this study are available on request from the corresponding author. The data are not publicly available due to data privacy.

## Abbreviations

The following abbreviations are used in this manuscript:

IMC	immune-mediated cholangitis
ICI	immune checkpoint inhibitor
IMH	immune-mediated hepatotoxicity
OS	overall survival
OR	odds ratio
CRP	C-reactive protein
HR	hazard ratio
NLR	neutrophil-to-lymphocyte ratio
CTLA-4	cytotoxic T-lymphocyte-associated antigen-4
PD-(L)1	programmed death-(ligand)1 receptor
imAE	immune-mediated adverse event
aPD-1 Ab	anti-PD-1 antibodies
aPD-L1 Ab	anti-PD-L1 antibodies
aCTLA-4 Ab	anti-CTLA4 antibodies
AST	aspartate transaminase
ALT	alanine aminotransferase
ALP	alkaline phosphatase
GGT	gamma-glutamyl transferase
PSC	primary sclerosing cholangitis
IHBD	intrahepatic bile duct
CT	computed tomography
MRCP	magnetic resonance cholangiopancreatography
ULN	upper limit of normal
PSL	prednisolone
MMF	mycophenolate mofetil
UDCA	ursodeoxycholic acid
IQR	interquartile range
WBC	white blood cell
CI	confidence interval
CBD	common bile duct
ECOG PS	Eastern Cooperative Oncology Group performance status
CTX	cytotoxic chemotherapy
MTT	molecular targeted therapy
ANC	absolute neutrophil count
ALC	absolute lymphocyte count
AEC	absolute eosinophil count
PLT	platelet
PLR	platelet-to-lymphocyte ratio
CTCAE	Common Terminology Criteria for Adverse Events
Nivo	nivolumab
Pem	pembrolizumab
Atezo	atezolizumab
ENBD	endoscopic nasobiliary drainage
MST	median survival time
